# An insertion mutation of ERBB2 enhances breast cancer cell growth and confers resistance to lapatinib through AKT signaling pathway

**DOI:** 10.1242/bio.047662

**Published:** 2020-01-24

**Authors:** Zi-Yan Yang, Liu Yang, Chun-Wei Xu, Xiao-Jia Wang, Lei Lei

**Affiliations:** 1 The Second Clinical Medical College of Zhejiang Chinese Medical University, Hangzhou, Zhejiang 310022, China; 2 Shanghai Dunlu Biomedical Technology Co. Ltd. Shanghai 201611, China; 3 Department of Pathology, Fujian Cancer Hospital, Fujian Medical University. No. 420, Fuma Road, Fuzhou, Fujian 350014, China; 4 Department of Chemotherapy, Zhejiang Cancer Hospital. No.1 Banshan East Street, Gongshu District, Hangzhou, Zhejiang 310022, China

**Keywords:** Breast cancer, ERBB2, Insertion mutation, Lapatinib, AKT

## Abstract

In clinical practice, some breast cancer (BC) patients carry a rare ERBB2 in-frame insertion (p. Pro780_Tyr781insGlySerPro) and are resistant to anti-ERBB2 therapy. To explore the potential procarcinogenic role of this ERBB2 mutation, we conducted the present study using BC cells overexpressing wild-type (WT) ERBB2 or P780-Y781 ERBB2 [mutated (MT)]. MDA-MB-231 and MCF-7 cells were transfected with the following plasmids using a lentivirus system: negative control (ERBB2-NC), WT ERBB2 overexpression (ERBB2-WT), and P780-Y781 ERBB2 overexpression (ERBB2-MT). P780-Y781 ERBB2 conferred significant resistance to lapatinib, as assessed by cell viability and colony counts. Analysis of the cell cycle showed that the P780-Y781 ERBB2 group showed an elevated proportion of cells in S, G2, and M phases compared with WT ERBB2 when exposed to lapatinib. Following lapatinib treatment, phosphorylated AKT (p-AKT) was strongly upregulated in the P780-Y781 ERBB2 group. Among ERBB2+ patients, the P780-Y781 ERBB2 group showed increased levels of p-AKT. Furthermore, the AKT inhibitor perifosine effectively suppressed lapatinib resistance, as indicated by the lapatinib inhibition curve and results of the colony formation assay, and decreased AKT phosphorylation. Altogether, we discovered a procarcinogenic mutation of ERBB2 that enhances BC cell growth through AKT signaling and causes resistance to lapatinib. Patients with this in-frame insertion mutation of ERBB2 should be recommended other therapeutic strategies apart from ERBB2 tyrosine kinase inhibitors, in particular lapatinib.

## Abstract (precis)

**Summary:** We report a novel insertion mutation of ERBB2, which enhances breast cancer cell growth and confers resistance to lapatinib. Combination of the AKT inhibitor with lapatinib can overcome this resistance.

## INTRODUCTION

Breast cancer (BC) is one of the most common and most fatal female malignancies (Sharma, 2019; Tao et al., 2019). Approximately 90% of BC deaths are attributed to metastatic progression accompanied by drug resistance (Maajani et al., 2019; Yeh et al., 2015; Lee et al., 2012; Ahn et al., 2007). Thus far, BC therapeutics have advanced significantly, especially targeted approaches, such as estrogen receptor (ER) and progesterone receptor (PR) targeting, as well as human epidermal growth factor receptor 2 (ERBB2 or HER2) targeting. ERBB2 is an important factor during the onset and progression of BC (Prat et al., 2019; Christgen et al., 2019). It is a preferred dimerization role among ERBB family for its high catalytic activity. Dimerization causes autophosphorylation of the tyrosine kinase domains. ERBB2 mediates various downstream carcinogenic signals, such as PI3K/Akt, Raf/MAPK/Erk, Notch, and STAT3 signaling (Mitsuda et al., 2018; Kebenko et al., 2015; Martín-Pérez et al., 2014; Mishra et al., 2012; Shin-Kang et al., 2011). Among invasive BCs, approximately 30% of cases exhibit amplified/overexpressed ERBB2, and ERBB2 is associated with a more aggressive phenotype and a poor prognosis (Lee et al., 2018). Generally, tyrosine kinase inhibitors (TKIs) that target ERBB2 can exert satisfactory treatment effects at an early stage. Among all targeted medications, lapatinib is one of the most effective dual TKIs, which interrupts both ERBB2 and EGFR pathways (Spector et al., 2005; Vogel et al., 2010). It is frequently used alone or in combination with other antitumor agents, such as trastuzumab, capecitabine, or letrozole (Vogel et al., 2010; Zhou et al., 2009; Greil et al., 2011; Paroni et al., 2012). However, many patients with metastatic BC do not respond to lapatinib or gradually develop drug resistance along with disease progression. At present, the mechanisms underlying lapatinib resistance are poorly understood. In our pilot clinical survey, we were interested in cases that had undergone next generation sequencing (NGS) and were found to carry a novel mutation in ERBB2. These patients commonly had an in-frame insertion mutation (c.2339_2340-ins-CGGCTCCCC, p. Pro780_Tyr781insGlySerPro) and exhibited lapatinib resistance. To explore the potential procarcinogenic role of this ERBB2 mutation, we conducted the present study using BC cells overexpressing wild-type (WT) or P780-Y781 ERBB2.

## RESULTS

### Clinical characteristics and spectrum of ERBB2 mutations in BC patients

**Fig. 1.**
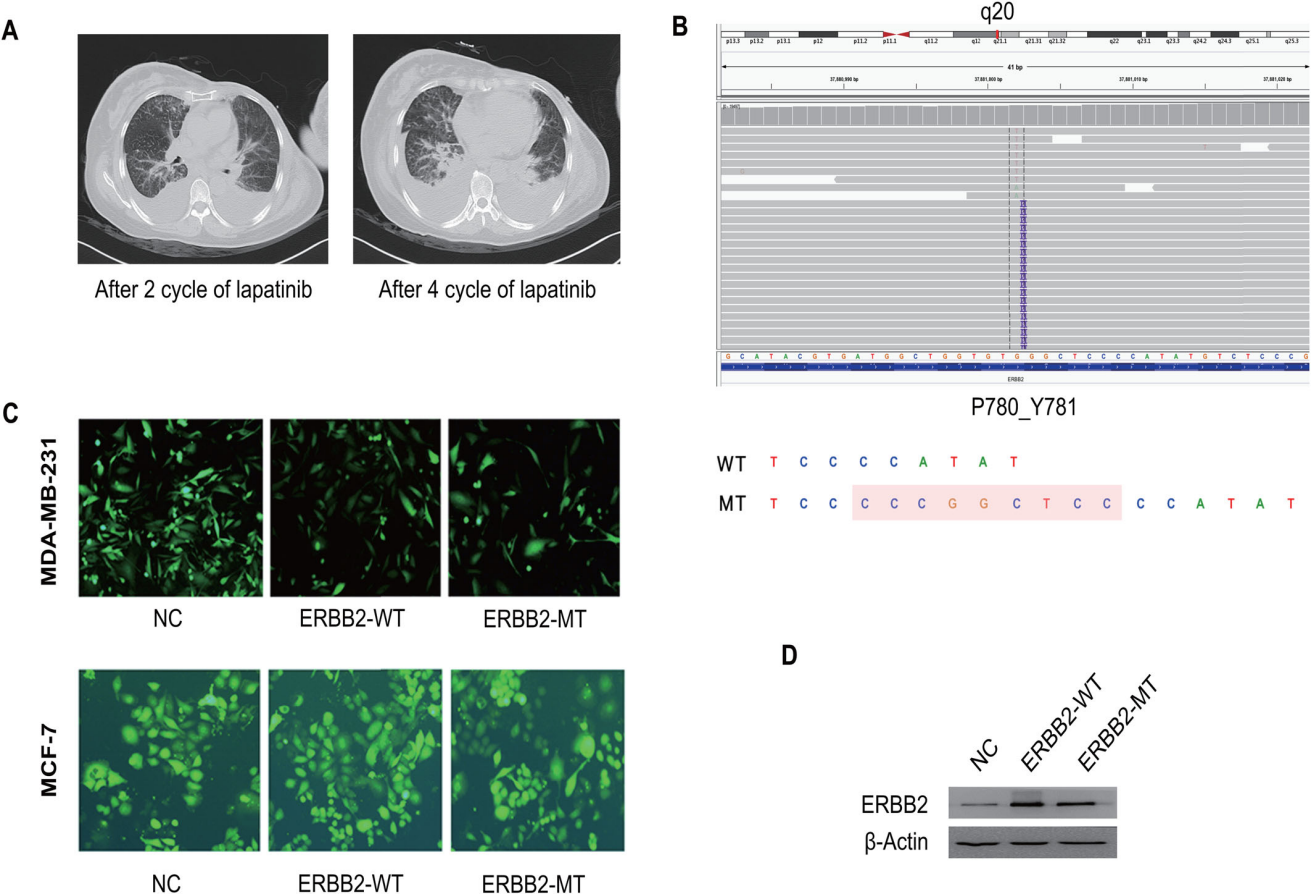
**Clinical characteristics and spectrum of ERBB2 mutations in breast cancer patients and construction of ERBB2 cell lines.** (A) Left: follow-up computed tomography (CT) image taken 2 months after initiation of lapatinib treatment, with significant pulmonary metastasis in multiple regions after two cycles of lapatinib treatment. Right: follow-up CT images taken at 4 months after initiation of lapatinib treatment, with more significant pleural effusion. (B) The gene sequencing result of the tumor after 4-month treatment with lapatinib (whole blood sample by means of ctDNA detection). The insertion mutation (c.2339_2340-ins-CGGCTCCCC, p.P780_Y781insGSP) was found in ERBB2 exon 20. The diagram of insertion mutation of ERBB2. Wild type, WT; P780-Y781 mutation, MT. (C) Three groups of lentiviruses were added to the MDA-MB-231 or MCF-7 culture to the validate the transfection efficacy (through observing the fluorescence under a fluorescence microscope). (D) The expression level of ERBB2-WT and ERBB2-MT were determined by western blot.

A 55-year-old female was diagnosed with stage IV breast cancer following axillary lymph node puncture in June 2016. Immunohistochemical results were as follows: ER (−), PR (−), and ERBB2 (−). She received surgery after four cycles of neoadjuvant chemotherapy. Two years later, contralateral breast and pulmonary metastasis occurred in March 2018. The postoperative pathological result was ER−, PR−, and ERBB2 (3+). Therapy with lapatinib and paclitaxel was given immediately. Meanwhile, the disease progressed rapidly with increased pleural effusion and aggravation of dyspnea. After four cycles of therapy, the patient developed acquired resistance to lapatinib (Fig. 1A). A tumor biopsy was sent for genomic profiling using an extensively validated hybrid-capture-based NGS diagnostic assay. Interestingly, the tumor was found to harbor a rare intragenic alteration in ERBB2, which resulted in the insertion mutation of exon 20 (Fig. 1B). The presence of p.Pro780_Tyr781insGlySerPro was confirmed by an independent clinical NGS assay (presented in Integrative Genomics Viewer, Burning Rock Biotech, Guangzhou, China).

### Construction of stable cell lines

To study whether the ERBB2 exon 20 insertion mutation imparts differential drug sensitivities to lapatinib based on TKIs, we engineered and expressed the mutation in MDA-MB-231 and MCF-7 cells. Cells transfected with mutant (p.P780_Y781insGSP) (ERBB2-MT), WT ERBB2 (ERBB2-WT), and the control (ERBB2-NC) are shown in Fig. 1C. After screening with puromycin, the expression levels of WT ERBB2 and P780-Y781 ERBB2 were determined by western blotting (Fig. 1D).

### Mutant ERBB2 confers resistance to lapatinib and is related to AKT signaling

**Fig. 2.**
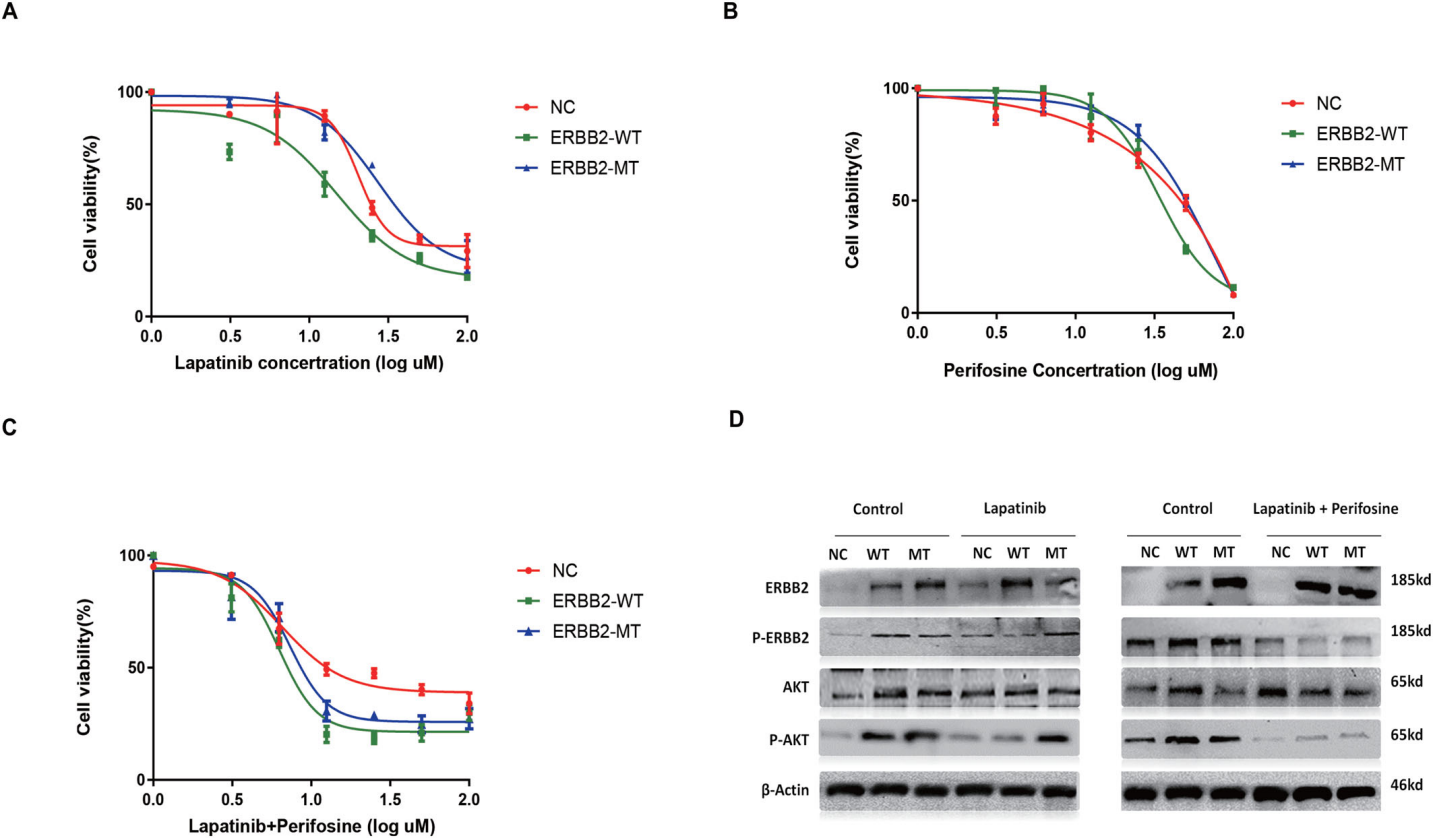
**Mutant ERBB2 confers a resistance to lapatinib and is related to AKT signaling pathway.** (A) Overexpression of ERBB2-MT enhances the lapatinib resistance (at 12.5 and 25 μM in comparison with ERBB-WT, both *P*<0.001). (B) The inhibition curve of AKT inhibitor perifosine. (C) Perifosine blocked shift up of the ERBB2-MT curve under (versus ERBB2-WT) under lapatinib stress. (D) AKT signaling pathway is associated with the ERBB2-MT mutation triggered resistance. Western blotting results confirmed that both ERBB2 strains had a significant increase in ERBB2 (as well as p-ERBB2) expression. Under lapatinib treatment, p-AKT expression was strongly upregulated in the ERBB2-MT group. Combination of the perifosine suppresses the AKT pathway.

First, we examined the effect of P780-Y781 ERBB2 on lapatinib inhibition of MCF-7 cell proliferation. In this experiment, the IC_50_ values of lapatinib for WT ERBB2 and P780-Y781 ERBB2 were determined to be 14.07±1.85 µM and 29.24±7.44 µM, respectively. Overexpression of P780-Y781 ERBB2 enhanced lapatinib resistance compared with WT ERBB2 (t=3.96, *P*=0.007) (Fig. 2A). As expected, the P780-Y781 ERBB2 group showed increased cell viability at lapatinib concentrations ranging from 12.5 to 25 μM (*F*=66.06, *P*<0.001), while the WT ERBB2 group showed no significance differences compared with the control. To probe the lapatinib resistance-associated mechanisms, we observed potentially carcinogenic signaling pathways, and only AKT signaling was found to be influenced by the ERBB2 mutation. Western blotting confirmed that both ERBB2-overexpressed groups exhibited a significant increase in the expression of ERBB2 (as well as p-ERBB2) in the control group. Following treatment with lapatinib, p-AKT expression was strongly upregulated in the P780-Y781 ERBB2 group (Fig. 2D, left). ERK and MEK pathways were not influenced by ERBB2 expression or polymorphism (data not shown).

To support the significance of AKT signaling, the AKT inhibitor perifosine was used. As shown in our pilot experiment (Fig. 2A,B), the IC_50_ values of perifosine for cells transfected with WT-ERBB2 and P780-Y781 ERBB2 were 33.86±1.36 µM and 54.96±7.04 µM, respectively. When cells were treated with lapatinib and perifosine, the IC_50_ values of WT ERBB2 and P780-Y781 ERBB2 decreased to 6.32±0.38 μM and 6.95±1.24 μM, respectively (t=1.04, *P*>0.05) (Fig. 2C). The inhibition curves of the P780-Y781 ERBB2 and WT ERBB2 groups showed that the inhibition of cell growth was significantly increased following treatment with both lapatinib and perifosine. Compared with lapatinib alone, AKT inhibition combined with lapatinib significantly enhanced the sensitivity to lapatinib in P780-Y781 ERBB2 cells (t=5.91, *P*<0.001). Western blotting confirmed that the phosphorylation of AKT and ERBB2 was not strongly activated following treatment with lapatinib and perifosine. Compared with lapatinib alone, AKT signaling in cells transfected with P780-Y781 ERBB2 was inhibited (Fig. 2D, right).

### P780-Y781 ERBB2 enhances colony-formation ability

**Fig. 3.**
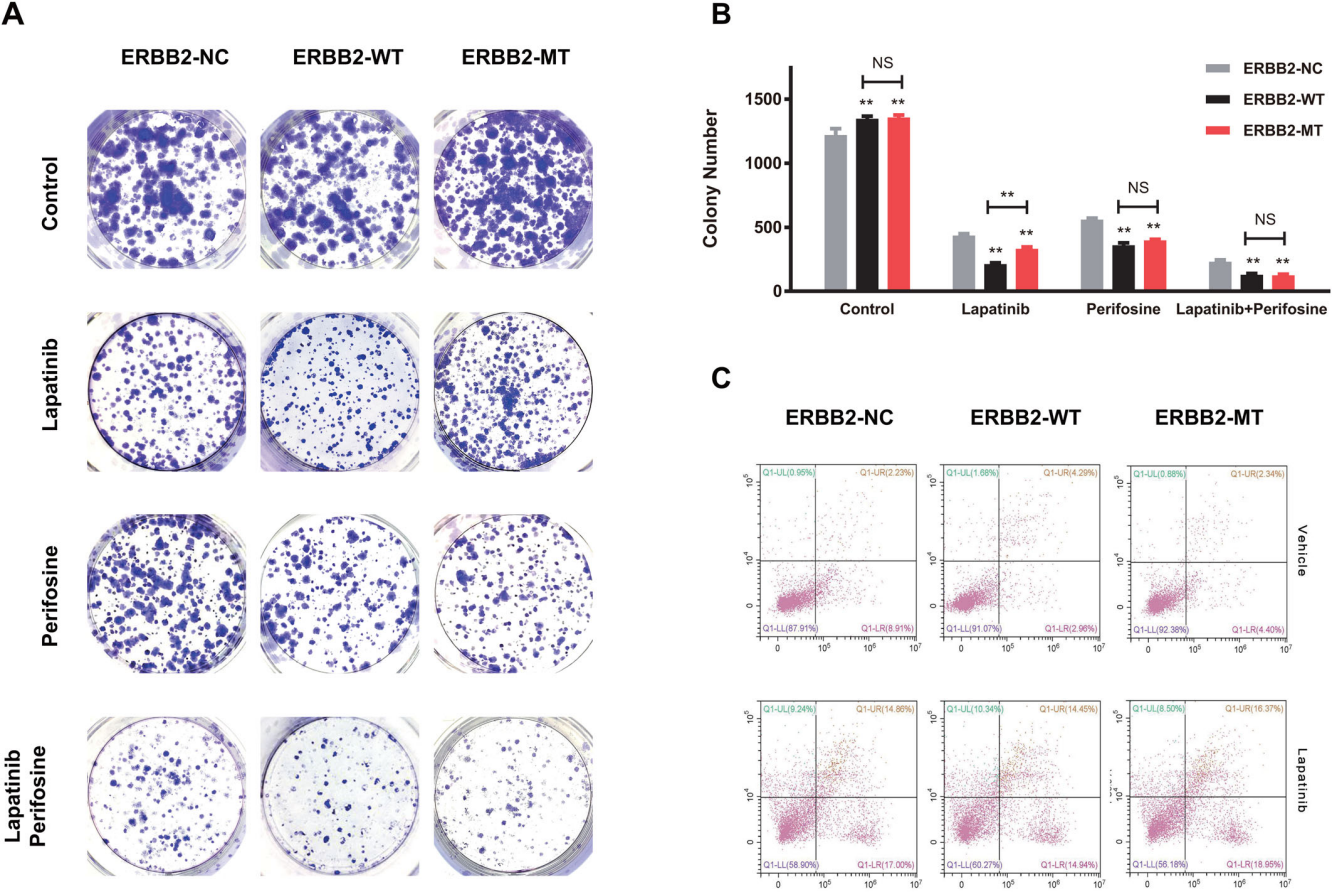
**P780-Y781 ERBB2 enhances colony-formation ability and but does not impact cell apoptosis.** (A) Mutant ERBB2 dramatically elevated the colony counts. Typical photos of colony staining of three groups under vehicle, lapatinib, perifosine and lapatinib+perifosine. (B) The colony counts in the ERBB2-NC and ERBB2-WT groups. ERBB2-MT dramatically elevated the colony counts in comparison with WT (t=7.01, *P*<0.05). However, under both lapatinib and perifosine stress, colony formation ability in the ERBB2-MT group and ERBB2-WT group were similar. (C) Three groups of cells showed similar apoptosis levels at normal condition and under lapatinib stress (*F*=4.05, *P*>0.05).

We further tested the potential enhancement of the anti-tumor effects of lapatinib in cells transfected with P780-Y781 ERBB2 when AKT signaling was blocked by treatment of perifosine. Next, the colony-formation ability following treatment with lapatinib stress explored. Similarly, WT ERBB2 (212±16.16) overexpression significantly impacted *in vitro* colony formation, while P780-Y781 ERBB2 (326±36.16) exhibited an increased number of colonies compared with WT ERBB2 (t=7.01, *P*<0.001). However, following treatment with perifosine, there were no significant differences between the WT ERBB2 (359±34.69) and P780-Y781 ERBB2 (397±15.39) groups (t=1.73, *P*>0.05). Following treatment with both lapatinib and perifosine, colony formation ability in cells transfected with P780-Y781 ERBB2 (125±14.17) and WT ERBB2 (129±17.78) was significantly decreased (Fig. 3A). Our results also showed that perifosine significantly enhanced the inhibitory effects of lapatinib on clonogenic survival of cells with the P780-Y781 mutation (t=12.58, *P*<0.001) (Fig. 3B).

### Apoptosis and the cell cycle

**Fig. 4.**
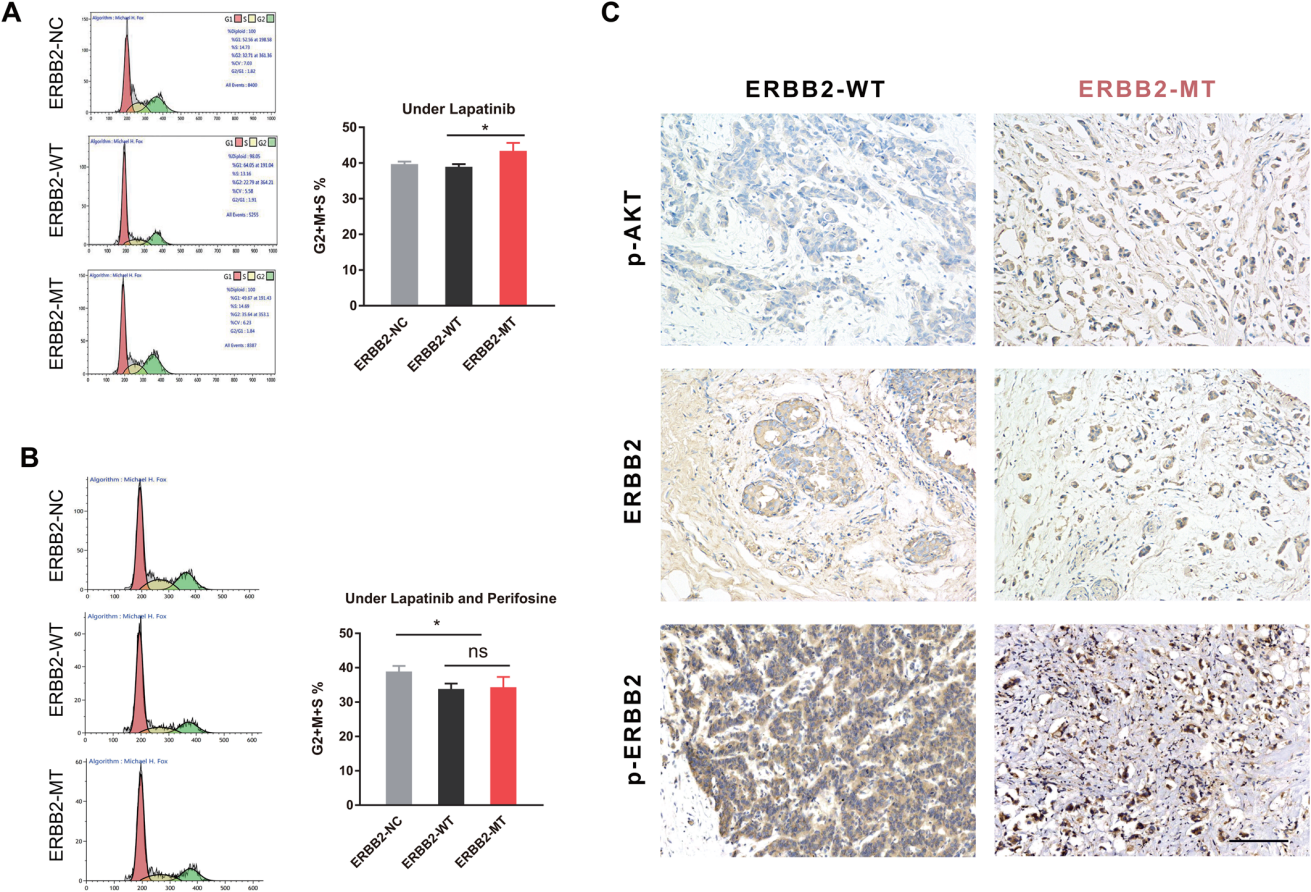
**ERBB2-MT group has an elevated proportion of (S+G2+M) stages and the ERBB2-MT tumor has a higher p-AKT level.** (A) In cycle distribution, the ERBB2-MT group showed an elevated proportion of (S+G2+M) stages compared with ERBB2-WT when exposed to lapatinib (t=14.97, *P*<0.05). (B) Under lapatinib+perifosine exposure, the G2+S+M proportion was significantly decreased in the ERBB2 overexpressed groups compared with NC, while no difference was found between WT and MT (t=0.25, *P*>0.05). (C) Immunohistochemistry staining of tumor sections from two groups of ERBB2+ BC patients. Both groups of patients had a clear positive expression of ERBB2 (and p-ERBB2), while the mutant group exhibited a dramatically higher expression level of p-AKT.

We also investigated the rate of apoptosis and the cell cycle distribution in different ERBB2 polymorphism groups. The three groups of cells showed similar levels of apoptosis in normal conditions and under lapatinib stress (*F*=4.05, *P*>0.05) (Fig. 3C). In terms of cell cycle distribution, P780-Y781 ERBB2 showed an increased number of cells in S, G2 and M phases of the cell cycle compared with WT ERBB2 when exposed to lapatinib (t=14.97, *P*<0.05) (Fig. 4A). Thus, mutant ERBB2 might enhance lapatinib resistance by increasing cell growth via proliferation, colony formation, and increased entry into the cell cycle. Interestingly, treatment with perifosine and lapatinib resulted in a significant decrease in the number of cells in S, G2 and M phases of the cell cycle in the ERBB2 overexpressed groups when compared with ERBB2-NC (*F*=4.93, *P*<0.05); no difference was found between the WT and the mutant (t=0.25, *P*>0.05) (Fig. 4B), suggesting that inhibition of AKT signaling may also influence ERBB2 signaling, jointly impacting the cell cycle under lapatinib stress. Together, overexpression of the ERBB2 mutation is significantly associated with proliferation; the P780-Y781 ERBB2 mutation may promote BC cell growth and lapatinib resistance through AKT signaling.

### Increased AKT activation in clinical ERBB2+ tumor sections

Finally, in support of the above results, we conducted IHC of tumor sections from two groups of ERBB2+ BC patients. Both groups exhibited clear expression of ERBB2 (and p-ERBB2), while the mutant group exhibited dramatically increased levels of p-AKT (Fig. 4C). Altogether, our results suggest that mutant ERBB2 may promote BC cell growth and lapatinib resistance through AKT pathway activation.

## DISCUSSION

In the present study, we addressed the potential reason why a proportion of BC patients exhibit lapatinib resistance clinically. In our pilot survey, we observed that some ERBB2+ BC patients experienced lapatinib treatment failure and rapid disease progression; this was frequently observed for those with an in-frame insertion mutation of ERBB2 [NM_004448.3, ERBB2: c.2339_2340-ins-CGGCTCCCC (p. Pro780_Tyr781insGlySerPro)]. The WT sequence is TCC-(780)-CCA-TAT; based on NGS the mutant sequence was determined to be TCC-(780)-CCCGGCTCCCCA-TAT. Therefore, it is reasonable to assume that this ERBB2 mutation may confer strong carcinogenicity, especially in terms of *in vivo* resistance to lapatinib.

ERBB2 is a widely reported oncogenic driver that is overexpressed in approximately 30% of BC cases and commonly leads to chemoresistance (D'Alesio et al., 2019; Yu and Hung, 2000). Previous studies have shown that ERBB2 mutation can impact the response to targeted BC therapy (Chumsri et al., 2015; Shih et al., 2015). For example, invasive lobular breast cancer (ILBC) in patients with an ERBB2 mutation may have a worse prognosis compared to counterparts without such a mutation (Ping et al., 2016). Acquired lapatinib resistance was also mediated by the mediator from ERBB2 (or from ER/ERBB2 cooperation) rather than ERBB2 loss or insensitivity of downstream signaling (Ginestier et al., 2007). Moreover, the gatekeeper mutation T798M in the active site of ERBB2 can cause acquired resistance to TKIs in BC therapy (Lu et al., 2019). In T798M mutant cases, substitution of the polar threonine with a bulky methionine at residue 798 can impact the binding affinity of TKIs. Mechanically, the L755S mutation may underlie the acquired resistance to ERBB2-targeted therapy and HER2 reactivation (Xu et al., 2017). The HER2 somatic mutation is an alternative mechanism to activate HER2 in BC, and these patients were sensitive to the irreversible HER2 inhibitor, neratinib (Bose et al., 2013). However, complete elucidation of the underlying mechanisms is still necessary. A possible mechanism that may explain our findings is that the inserted Gly-Ser-Pro can be phosphorylated and escape lapatinib inhibition. ERBB2 mutation may also be correlated with mutation of E-cadherin (CDH1), a glycoprotein that mediates adhesion between epithelial cells and suppresses cancer invasion. It has been reported that CDH1-mutated ILBC patients have a high frequency of ERBB2 gene mutations (23%) (Ross et al., 2013), which possibly results in BC relapse.

We revealed here that AKT signaling may be a key factor mediating P780-Y781 ERBB2-derived resistance. This is consistent with several published reports. For example, ERBB2-associated procarcinogenic activity can be suppressed via inactivation of the PI3K/AKT pathway (Liu et al., 2007; Croessmann et al., 2019; Carmona et al., 2016; Jegg et al., 2012). A recent study used isogenic knock-in ERBB2 mutations in ER+ MCF-7 cells and xenografts, and discovered that ERBB2 mutations can hyperactivate the HER3/PI3K/AKT/mTOR axis, leading to anti-estrogen resistance in ER+ BC (Croessmann et al., 2019). Additionally, it has been suggested that AKT inhibitors in combination with gemcitabine can be a strategy for the treatment of trastuzumab-resistant ERBB2+ BC (Wang et al., 2014). Further studies are needed in BC cell lines and animal models to verify the sensitizing activity of AKT inhibitors, and to assess the clinical efficacy of the AKT inhibitor and lapatinib combination using neratinib as a control (Ma et al., 2017).

Finally, some limitations exist in the present study. First, we mainly investigated the influence of P780-Y781 ERBB2 on *in vitro* cell functions even though this phenomenon was originally observed clinically; we have not performed *in vivo* studies. Our conclusions will require verification in tumor-bearing animal models. Our results suggest that the P780-Y781 ERBB2-derived resistance to lapatinib was mainly associated with cell proliferation and not due to its anti-apoptotic role. Although many experiments have been carried out, significant differences only occasionally appeared; in most cases, we hardly observed any anti-apoptotic function of P780-Y781 ERBB2, which will require further investigation. However, it has previously been reported that the anti-apoptotic function of ERBB2 is important in ERBB2-mediated resistance (Yu and Hung, 2000; Ginestier et al., 2007). Although overexpression of mutated ERBB2 (especially p. Pro780_Tyr781insGlySerPro in the present study) may exert different downstream effects compared with WT ERBB2 overexpression, it is impossible to determine the relationship between mutated ERBB2 and apoptosis.

### Conclusions

In conclusion, we discovered a procarcinogenic mutation of ERBB2 that enhances BC cell growth through the AKT signaling pathway and causes resistance to lapatinib. Patients with the ERBB2 c.2339_2340 insertion should be recommended other therapeutic strategies apart from ERBB2 TKIs, and in particular, lapatinib.

## MATERIALS AND METHODS

### Patients

ERBB2 exon 20 mutations were identified in BC patients at the Zhejiang Cancer hospital as part of a routine genotyping effort. The methods of detection included NGS technology. The patients provided written informed consent, and the study was performed in accordance with the Declaration of Helsinki and was approved by the Institutional Review.

### Cell culture

The breast cancer cell lines MDA-MB-231 and MCF-7, as well as 293T cells were obtained from the American Type Culture Collection (Manassas, VI, USA). Cells were cultured in RPMI-1640 medium (Invitrogen, Thermo Fisher Scientific, USA) containing 10% fetal bovine serum, 100 U/ml penicillin, and 100 mg/ml streptomycin (Invitrogen, Thermo Fisher Scientific). The cells were incubated at 37°C with 5% CO_2_ and saturated humidity; only cells in logarithmic growth phase were used for subsequent experiments.

### Expression constructs and cell culture

The following stable cell lines were constructed: negative control (ERBB2-NC), WT ERBB2 overexpression (ERBB2-WT), and P780-Y781 ERBB2 overexpression (ERBB2-MT). The WT ERBB2 sequence was amplified by PCR at the restriction sites BamHI/AgeI and recombined into the vector GV358_Ubi-MCS-3FLAG-SV40-EGFP-IRES-puromycin.

The mutation site of ERBB2 was p.P780_Y781insGSP, and the corresponding specific primers (forward, AGGTCGACTCTAGAGGATCCCGCCACCATGGAGCTGGCGGCCTTGTGCCGCTGG, reverse, TCCTTGTAGTCCATACCCACTGGCACGTCCAGACCCAGGTACTCTGG) were designed by GENE company (Shanghai, China). Similarly, the P780-Y781 ERBB2 sequence was amplified at the restriction sites BamHI/AgeI and recombined into the vector GV492_Ubi-MCS-3FLAG-CBh-gcGFP-IRES-puromycin. At the same time, a vector containing only CopGFP sequence, pHelper. CopGFP, was constructed as a control vector. The recombined vector was verified by sequencing and then packed by lentivirus. Three plasmids with packaging mix were used to transfect 293T cells, and viruses were collected after 48 h followed by titer determination. Three groups of lentiviruses were added to MDA-MB-231 and MCF-7 cells to validate transfection efficacy by fluorescence microscopy. After screening by puromycin, the expression levels of WT ERBB2 and P780-Y781 ERBB2 were determined by western blotting.

### Cell viability analysis

Cell viability was determined using the CCK8 assay. First, the three groups of cells were seeded on 96-well plates at 40,000 cells/well. Following attachment, cells were incubated with different concentrations of lapatinib (S2111, Selleckchem, Houston, TX, USA) and/or perifosine (1.5–100 μM APExBIO, Houston, TX, USA) and cultured for 48 h. CCK8 agent (Dojindo Molecular Technologies, Inc., Japan) was added, and after 2 h, the absorbance at 450 nm was measured using a microplate reader (Bio-Rad Laboratories, Hercules, CA, USA).

### Western blot analysis

Cells were collected and homogenized in lysis buffer with protein inhibitor and 1 mM PMSF (Beyotime Institute of Biotechnology, Jiangsu, China) for 30 min on ice. The lysates were centrifuged at 12,000× ***g*** at 4°C for 10 min, supernatants were collected, and protein concentration was measured using the BCA assay (Beyotime Institute of Biotechnology). For each group, protein samples were separated using 10% SDS-PAGE and transferred onto polyvinylidene fluoride membranes. Membranes were blocked in TBST-milk and incubated with the following primary antibodies: rabbit anti-ERBB2 (18299-1-AP); mouse anti-AKT (60203-2-lg); mouse anti-ERK (66192-1-lg); mouse anti-p-AKT (66444-1-lg; phospho-S473; Proteintech, Wuhan, China), rabbit anti-p-ERK (4370T; phospho-Thr202/Tyr204; Cell Signaling Technology, Danvers, MA, USA); rabbit anti-p-ERBB2m (GTX133439; phosphor-Ty1248; GeneTeX, Shanghai, China), and mouse anti-β-actin (Proteintech). After three washes with TBST, membranes were incubated with the corresponding secondary antibodies (goat anti-mouse or -rabbit; Proteintech) and the bands were visualized using an enhanced chemiluminescence kit (Beyotime Institute of Biotechnology).

### Colony-forming assay

Cells transfected with the different plasmids were seeded in six-well plates at a density of 500 cells/well and treated with different concentrations of lapatinib, perifosine, or a combination of lapatinib and perifosine for 48 h. Cells were cultured for an additional 2 weeks. Colonies (>50 cells) were stained with Crystal Violet and each well was photographed. The number of colonies was counted under an inverted microscope.

### Cell cycle analysis

The different groups of MCF-7 cells were cultured at a density of 1×10^5^ cells/well in six-well plates and treated with lapatinib or lapatinib and perifosine for 48 h. Cells were washed with cold phosphate-buffered saline (PBS), treated with 70% ethanol at 4°C overnight, and the cell density was diluted to 5×10^5^ cells/ml. Cells were stained with Propidium Iodide (50 µg/ml, MultiSciences, Hangzhou China) in the presence of RNase A (100 µg/ml), followed by flow cytometry (Beckman Coulter, Brea, CA, USA) to determine cell cycle distribution. Each experiment was performed in triplicate.

### Analysis of apoptosis

The different groups of MCF-7 cells were cultured (1×10^5^ cells/well) and treated with lapatinib (at the IC_50_ concentration) for 48 h. Cells were then collected, washed with ice-cold PBS, and the cell concentration was adjusted to 5×10^5^ cells/ml using staining buffer. Cell apoptosis was assayed by staining with 5 μl Annexin V-PE and 7-AAD (MultiSciences, China) for 30 min at room temperature in the dark, and the proportion of apoptotic cells was determined with a flow cytometer (Beckman Coulter). Each experiment was performed in triplicate.

### Immunohistochemistry (IHC)

Given that AKT signaling might be activated in mutant ERBB2 BC cells, we performed IHC to analyze AKT activation in clinical tumor samples. ERBB2-positive BC patients were divided into two groups: WT ERBB2 and P780-Y781 ERBB2. Three tumor samples in each group were dehydrated and embedded in paraffin. The 5-μm paraffin sections were deparaffinized with xylene and anhydrous alcohol and washed with distilled water, followed by antigen retrieval. Next, sections were placed in 3% hydrogen peroxide and incubated for 25 min at room temperature. Tissues were blocked with 3% bovine serum albumin at room temperature for 30 min, primary antibodies were added dropwise to sections, and the sections were incubated overnight. Subsequently, sections were washed three times with PBS and the corresponding horseradish peroxidase-labeled secondary antibodies were added. After 1 h of incubation and three washes, DAB solution was added for 1 min. The DAB solution was washed away and sections were counterstained with Hematoxylin for 3 min. Following dehydration, each section was examined under a microscope to observe the following proteins: ERBB2, p-ERBB2, AKT, and p-AKT.

### Statistical analysis

All continuous quantitative data were compared using the independent-sample Student's *t*-test (between two groups) or one-way analysis of variance (more than two groups). Data analysis was performed using GraphPad Prism software (La Jolla, CA, USA). Data are expressed as mean±s.d. For all the presented data, *P*<0.05 was considered statistically significant.
